# Formulation Design, Optimization, and Evaluation of Solid Lipid Nanoparticles Loaded With an Antiviral Drug Tenofovir Using Box–Behnken Design for Boosting Oral Bioavailability

**DOI:** 10.1155/2024/5248746

**Published:** 2024-12-31

**Authors:** Sri Rekha M., Sangeetha S.

**Affiliations:** Department of Pharmaceutics, SRM College of Pharmacy, SRMIST, Kattankulathur, Chennai, Tamil Nadu, India

**Keywords:** Compritol 888 ATO, nanocarriers, risk assessment, solid lipid nanoparticles, tenofovir

## Abstract

**Purpose:** The current study aimed to improve the oral bioavailability of tenofovir (TNF), an antihuman immunodeficiency viral (HIV) drug, by integrating it into solid lipid nanoparticles (SLNs), an emerging lipid formulation.

**Method:** The suggested SLNs were generated utilizing the microemulsion process, using Compritol 888 ATO. A Box–Behnken experimental design was attempted to analyze the impact of critical quality attributes (CQAs), such as lipid and surfactant content and homogenization duration on response metrics such as particle size (PS) and percentage entrapment. The prepared SLNs were assessed for entrapment efficiency, zeta potential (ZP), PS, polydispersity index, and in vitro drug release. Moreover, ex vivo permeation tests employing goat intestinal sacs, solid-state characterization by DSC and PXRD, surface morphology by SEM, and in vivo pharmacokinetic evaluation using albino Wistar rats were conducted.

**Results:** The research findings demonstrated that a formulation composed of 5.5% lipid and 2% surfactant had a comparatively smaller PS (449.90 ± 4.79 nm), a narrow size distribution (0.304 ± 0.004), and strong stability with an entrapment efficiency of 83.13 ± 6.34% and a negative ZP (−18.10 ± 2.35 mV). According to in vitro drug release experiments, first-order kinetics were followed and 99% of the medication was released over the time course of 24 h. In albino Wistar rats, an in vivo pharmacokinetic analysis of the optimized formulation (F10) showed a 12.4-fold improvement in bioavailability over pure TNF solution.

**Conclusion:** This study suggests the potential of SLNs in overcoming bioavailability issues, particularly low permeability, gut metabolism, and P-gp efflux transport.

## 1. Introduction

Tenofovir (TNF) is a drug used in highly active antiretroviral therapy (HAART), a treatment for human immunodeficiency virus (HIV)/AIDS [1–3]. It is a nucleotide reverse transcriptase inhibitor (NtRTI) [4], and patients in the pediatric and adolescent age range of 12–18 years old can use it safely and effectively [5]. Despite this, its low log *p* value (−1.6) [6] and the presence of free hydroxyl groups in its chemical composition, which easily ionize in gut pH and reduce its permeability, limit its oral bioavailability. Furthermore, it is susceptible to metabolism by the gut wall esterases and P-glycoprotein (P-gp) efflux transport system [7–9]. TNF disoproxil fumarate (TDF), a prodrug version, is presently used to increase its bioavailability [10]. While TDF's pharmacokinetic profile and antiviral activity have been demonstrated to be better than those of its parent drug TNF, its systemic bioavailability is still very low—it is only 25% bioavailable, according to reports [11]. Moreover, chronic TDF use has been connected to renal and bone damage [12–14]. TNF reformulation may result in substantial cost savings by lowering the dosage needed while preserving effectiveness, guaranteeing stability across a broad pH range, reducing enzymatic degradation, and preventing P-gp-driven efflux.

To address the issues mentioned earlier, a new approach is being developed in the form of a colloidal carrier system that can overcome gut metabolism and efflux transport. Among the various options available, solid lipid nanoparticles (SLNs) have garnered considerable interest due to their numerous advantages such as small particle size (PS) [15], improved drug stability, increased bioavailability [16], protection against enzymatic metabolism, ability to carry both hydrophilic and lipophilic drugs [17], ease of large-scale production [18, 19], and more. SLNs provide the advantages of other colloidal carrier systems while avoiding their drawbacks. Unlike polymeric nanoparticles (NPs), they are biodegradable and biocompatible, and unlike emulsion formulations, they have sustained release effects because the drug is immobilized within solid lipids. Moreover, SLNs have greater chemical and physical stability than liposomes [20]. Moreover, SLNs circumvent hepatic and enzymatic metabolism and lower P-gp efflux by being absorbed via the lymphatic system [21]. Therefore, this may be used as an advantage to increase the oral bioavailability of TNF.

SLNs are submicron colloidal carriers composed of solid lipids that are disseminated in an aqueous surfactant solution. The use of a solid lipid instead of a liquid lipid is an advantage as it has better control over the drug release and can also protect the encapsulated drug from chemical degradation and enzymatic metabolism. Solid lipids, which are part of the biological system and generally recognized as safe (GRAS), such as fatty acids like stearic acid (SA), triglycerides like tristearin and tripalmitin, hard fats like Witepsol, waxes like cetyl palmitate (CP) and beeswax, and acylglycerols like glyceryl monostearate (GMS), are major components of SLN formulations [22, 23]. Different technologies used to fabricate SLNs are high-shear homogenization, high-pressure homogenization (HPH) (cold and hot homogenization), solvent emulsification, solvent evaporation, ultrasound, and microemulsion methods [24, 25].

TNF is a hydrophilic drug having high solubility (13.4 mg/mL) and low permeability (log P of −1.6) [26], classified as Class III in the Biopharmaceutical Classification System (BCS) [27]. Its limited permeability is known to cause low oral bioavailability. Although scientists have produced prodrugs [28] and polymeric NPs [29] to boost oral bioavailability, none have proposed employing SLNs. However, because hydrophilic medicines often partition in the aqueous phase during manufacture, developing SLNs that can efficiently encapsulate these medications is difficult [24]. By carefully choosing the appropriate lipids, surfactants, and their composition, this can be avoided. Moreover, examining the effects of different formulations and process factors on the desired attributes of the lipid nanocarriers is crucial to achieving the optimal SLN formulation.

This study's main objective was to create and enhance TNF-loaded SLNs in order to increase their oral bioavailability using the design of experiments (DOE) process. In order to do this, the formulation was optimized using a Box–Behnken design (BBD), and the impact of various processing and formulation parameters on crucial quality measures including PS and percentage entrapment efficiency (%EE) of the SLN was investigated. A number of characterization studies were conducted to ascertain the SLNs' shape, size, %EE, and drug release kinetics. Moreover, studies on the excised gut sac and in vivo pharmacokinetics were carried out to enhance the comprehension of the process of oral absorption of SLNs loaded with TNF.

## 2. Materials and Methods

### 2.1. Materials

A complimentary sample of TNF and Compritol 888 ATO was received from MSN Laboratories Pvt. Ltd., Hyderabad, India. Sigma-Aldrich in Mumbai, India, supplied SA, GMS, cholesterol, poloxamer 188, poloxamer 407, and soya lecithin. Span 60, Tween 80, beeswax, and stearyl alcohol were purchased from Loba Chemie Pvt Ltd. in Mumbai, India. Merck Ltd., Mumbai, India, was the source of HPLC quality solvents such as methanol, acetonitrile, and water. The remaining chemicals and solvents were all of analytical grade.

### 2.2. Methods

#### 2.2.1. Lipid Phase Screening

While selecting lipids, the maximum solubility of a medication was taken into account. TNF solubility was assessed in a range of lipids, including SA, beeswax, glyceryl palmitostearate (GPS), cholesterol, stearyl alcohol, and GMS. To test for solubility, 1 g of melted fat that had been heated to 10 degrees Celsius over its melting point was combined with 5 mg of carefully weighed TNF. Then, a fraction of the medication (5 mg) was introduced immediately when the previous fraction was dissolved incrementally while being continually swirled at 100 rpm using an orbital shaking incubator until the drug was no longer able to dissolve in the molten lipids. Following a 24-h shaking period, the solubility was evaluated visually; the appearance of a turbid solution served as the endpoint, and the maximum amount of TNF dissolved in the lipid was recorded. The lipid with the highest drug solubility was chosen for additional formulation research because it was thought to have the highest drug loading capacity [30, 31].

#### 2.2.2. Surfactant Screening

The average diameter of the particles and the %EE of the resulting SLN dispersion by the surfactant system were used to determine which surfactant to use. Trial batches of SLNs were prepared using a variety of surfactants, such as Span 60, Tween 80, poloxamer 407, and poloxamer 188, in order to determine the best surfactant system. In a nutshell, 3% lipid (Compritol 888 ATO) was taken and melted at 70°C. Similarly, an aqueous phase was produced by dissolving 2% surfactant in the required volume of distilled water at the same temperature. Both phases were mixed and agitated at 500 rpm for 10 to 15 minutes in order to create a uniform emulsion. Following a high-speed homogenizer run at 20,000 rpm for 10 min, the resultant emulsion was assessed for PS and %EE. The highest entrapment and smallest particle diameter were the criteria used to select the surfactant of choice for further experiments [32, 33].

#### 2.2.3. Selection of Formulation Technique

HPH, high-speed homogenization, ultrasound, solvent emulsification and evaporation, microemulsion technology, and other methods are used in the formulation of SLN. In the current investigation, SLN with a chosen lipid (3% Compritol 888 ATO) and surfactant (1% poloxamer 188) were prepared using widely used procedures such as high-speed homogenization, solvent emulsification and evaporation, and microemulsion techniques. The evaluation of PS and % EE with the trial batches was the basis for choosing the formulation procedure [32, 34, 35].

#### 2.2.4. Drug and Excipient Compatibility Study

Compatibility tests were conducted using Fourier transform infrared spectroscopy (FTIR) to determine the drug's interaction with certain excipients. The drug, lipid, surfactant, and physical blend of drug and chosen excipients were held at 25°C, 60% ± 5%RH for 7 days. The infrared spectra of these samples were acquired in the 4000 − 400 cm^−1^ region, and they were compared to look for any notable changes [36–38].

#### 2.2.5. Quality by Design and Risk Assessment

**Figure 1:**
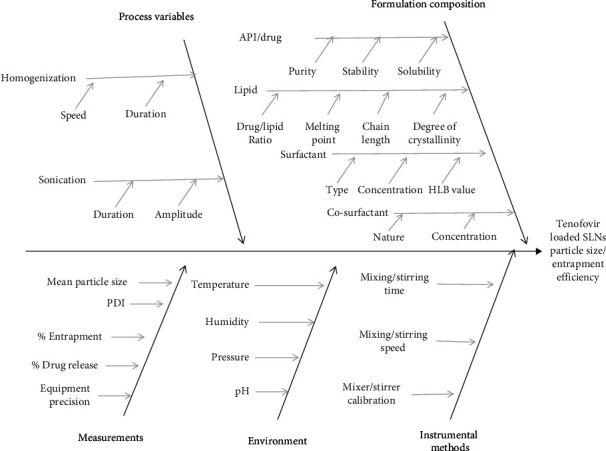
Ishikawa/fishbone diagram for risk assessment in development of TNF-loaded SLNs.

Critical process parameter (CPP) optimization was performed using the quality by design concept. The purpose of the Ishikawa/fishbone diagram for risk assessment is to pinpoint probable sources of product variability [39].  Table 1 presents several facets of the quality target product profiles (QTPPs) and critical quality attributes (CQAs), whereas Figure 1 illustrates the risk assessment for TNF-loaded SLN development using a fishbone layout.

#### 2.2.6. Experimental Design

Top-notch Box–Behnken Design (BBD) using Design-Expert software (STAT-EASE 360 trial edition), an optimization strategy comprising three variables and three levels with 13 experimental runs was attempted. To investigate the impact of lipid concentration (A), surfactant concentration (B), and homogenization time (C) on two responses, PS, and %EE, as well as their interaction, the variables were set at low, medium, and high levels. The current study's objective function was chosen to maximize EE while lowering PS. Analysis of variance (ANOVA) was used to assess the statistical reliability of the polynomial equations generated by Design-Expert software. The factor coding and experimental runs are shown in Table 2. Lipid levels were 1%, 5.5%, and 10% at low, medium, and high levels, respectively. Surfactant levels were 1%, 2%, and 3%, while homogenization times were 30, 60, and 90 min [33, 38, 40].

#### 2.2.7. Method of Preparation

Using the previously published microemulsion techniques [41–44], TNF-loaded SLNs were prepared using the selected lipid Compritol 888 ATO, the surfactant poloxamer 188, and soy lecithin as cosurfactants. Using the BBD, a total of 13 formulations were created by altering the quantities of lipid, surfactant, and homogenization time, as shown in Table 2. In summary, before adding TNF, the lipid phase (Compritol 888 ATO) was heated by 10°C over its melting point (70°C). The aqueous phase containing the cosurfactant (soya lecithin) and surfactant (poloxamer 188) was heated to the same temperature as the lipid phase. An o/w emulsion was produced by introducing the hot aqueous phase to the molten lipid phase and continuously swirling for 10–15 min at 500 rpm on a magnetic stirrer. Now, the hot emulsion was carefully poured into a beaker containing ice-cold water (8°C) in a dropwise manner under continuous homogenization for the duration given by BBD at 20,000 rpm and then subjected to probe sonication. After 10 minutes of probing sonication at 60% amplitude (Sonics Vibra™ Cell Ultrasonic Processor, 130 W, 20 kHz, USA), the generated SLNs with reduced particles to nanoscale were kept at 4°C for further analysis. After 10 minutes of probing sonication at 60% amplitude (Sonics Vibra™ Cell Ultrasonic Processor, 130 W, 20 kHz, USA), the generated SLNs with reduced particles to nanoscale were kept at 4°C for further analysis.

### 2.3. Characterization of Formulated TNF-Loaded SLNs

#### 2.3.1. PS, Polydispersity Index (PDI), and Zeta Potential (ZP)

The Nanoparticle Size Analyzer SZ-100Z, Horiba Ltd., Japan, was used to measure the average diameter of particles, PDI, and ZP of TNF-loaded SLNs. All formulations were diluted to an appropriate concentration with double distilled water prior to testing. The samples were analyzed by placing them in disposable cuvettes, and the findings for PS, PDI, and ZP were noted [45, 46].

#### 2.3.2. EE

The %EE of TNF-loaded SLNs was measured by an indirect method using a high-speed cooling centrifuge (Remi Instruments, Ltd., Mumbai, India) to assess unentrapped drugs in an aqueous medium. By deducting the amount of drug found in the supernatant from the total amount of drug used to make SLNs, the amount of drug entrapped in SLN was calculated. To put it briefly, a centrifuge tube containing a known volume of TNF-loaded SLN dispersion was centrifuged for 20 min at 4° C at 12,000 rpm. After appropriate dilutions, the supernatant was collected and tested for free drug content using a UV–visible spectrophotometer at 262 nm (*λ*_max_) [44]. The following formula is used to determine the %EE:

(1) $$\begin{array}{c} \%\text{ entrapment efficiency}=\frac{\text{total drug content}-\text{free drug}}{\text{total drug content}}\times 100. \end{array}$$

### 2.4. Optimization of Variables

Based on two quality parameters—PS and EE, the formulations made in accordance with the experimental design were examined and refined to determine the ideal ratios of lipid, surfactant, and homogenization duration. The trial version of STAT-EASE 360 was used to analyze statistical data using ANOVA, generate model equations, and produce contour plots for every response. The relationship between the independent and dependent variables was also established using three-dimensional (3D) surface plots. The desirability function of PS was set to minimal, and EE was adjusted to greatest in order to optimize the formulation. The optimal formulation was determined by the *p* value, and its validity was confirmed by the predicted *R*^2^ and ANOVA values [47].

### 2.5. In Vitro Drug Release Studies

In vitro release of TNF from SLN formulation was performed by dialysis membrane diffusion technique [48–50] using a dialysis membrane with a 12,000–14,000 Da molecular limit. In summary, the dialysis membrane was knotted at both ends and stretched over the diffusion glass vial after being immersed in the release media for the entire night. A defined volume of TNF-loaded SLN dispersion equivalent to 10 mg drug was placed in a dialysis tube and then inserted into a beaker containing 100 mL of diffusion medium. The mixture was held at 37 ± 0.5°C and swirled continuously at 100 rpm using a magnetic stirrer (REMI 2MLH). The dissolution analysis was conducted under two distinct conditions: initially, in simulated stomach fluid (pH 1.2) and then in simulated intestinal fluid (pH 6.8) during the final 22 hours. To keep the sink conditions, 2 mL of aliquots were taken out and replaced with fresh buffer at different time intervals of 0, 0.5, 0.75, 1, 2, 3, 4, 5, 6, 8, 10, 12, 15, 18, 21, and 24 h. A UV–visible spectrophotometer was used to evaluate each sample at 262 nm [51]. The amount of drug released was then used to calculate the cumulative percentage of drug release:

(2) $$\begin{array}{c} \text{amount release}=\frac{\text{test absorbance}\times \text{dilution factor}\times \text{vol}.\text{ of dissolution medium}}{\text{standard absorbance}\times 1000}\times \text{standard concentrat}i\text{on},\\ \%\text{ drug release}=\frac{\text{amount release}}{\text{total drug used in the formulation}}\times 100. \end{array}$$

### 2.6. Release Kinetics

To acquire a better understanding of the drug release mechanism from SLN formulation, the data from the in vitro drug release study were fitted into several kinetic models, including zero-order (cumulative% drug release vs. time), First-order (log% drug remaining vs. time), and Higuchi's models (cumulative% drug release vs. square root of time). The regression analysis of the linear curve produced by the previous plots was used to compute the values of *K* and *R*^2^. Then, the exact mechanism of drug release was determined from the release exponent value (*n*) of Korsmeyer–Peppas model (log % drug release vs. log time) [52–55].

### 2.7. Solid-State Characterization

#### 2.7.1. Differential Scanning Calorimetry (DSC)

The Exstar DSC7020, Hitachi HTG, Japan, was used to record DSC thermograms of pure drug TNF, physical mixture of drug and excipients, and optimal formulation (TF10). All samples were heated in an inert atmosphere created by flushing nitrogen gas with a flow rate of 40 mL per minute within a hermetically sealed aluminum pan under a constant temperature of 10°C/min from 30–300°C [56–58].

#### 2.7.2. Powder X-Ray Diffractometry (PXRD)

A Rigaku Miniflex 600, Rigaku Corporation, Japan, model x-ray diffractometer was used to obtain XRD patterns of TNF, physical mixture of TNF and Compritol 888 ATO, poloxamer 188, and improved SLN formulation. The generator had a voltage of 40 KV and a current of 15 mA, employing Cu as the anode substance. Every sample was put through CuK*α* radiation at a rate of 0.0200 deg each step over two angles ranging from 3° to 90°. The acquired PXRD patterns were compared to the pure drug's typical drug peak intensity [30, 59, 60].

#### 2.7.3. Scanning Electron Microscopy (SEM)

Utilizing the VEGA 3, SBH, TESCAN Brno S.R.O., Czech Republic, type scanning electron microscope, the optimized batch of TNF-loaded SLNs (TF10) was examined for surface characteristics and geometry. In brief, the sample was coated in gold and then examined under various magnifications at 15,000 V accelerating voltage [54, 61].

### 2.8. Ex Vivo Permeation Study Using Gut Sac Method

Using Franz's diffusion model, an intestinal permeability investigation of optimized TNF-loaded SLNs (TF10) was performed across goat intestines. The jejunal section of a freshly cut goat intestine was carefully isolated and cleaned using 0.98%w/v solution of sodium chloride to get rid of the mucus and lumen contents. The intestine specimen was positioned with its epithelial surface facing upward across the donor and receptor compartments of the Franz diffusing cell after soaking for an hour in saline water. Ten milligrams, or the equivalent, of a drug suspension and optimized TNF-loaded SLN dispersion (TF10) was placed in the donor chamber and wrapped in aluminum foil to avoid drying. Space in the receptor compartment was filled using 10 mL of pH 7.4 buffer, and the entire unit was held at 37 ± 0.5°C under constant magnetic stirring. An adequate sample was withdrawn at different time points (0, 0.25, 0.5, 1, 2, 3, 4, 6, and 8) over a period of 8 h and immediately substituted by an equal amount of new medium, and then, the samples were tested using a UV–visible spectrophotometer at 262 nm [62–66].

### 2.9. In Vivo Bioavailability Study

Institutional Animal Ethics Committee (IAEC), Hindu College of Pharmacy, Andhra Pradesh, India, approved the study protocol (IAEC-HCOP/2022/07). Eighteen healthy albino Wistar rats weighing 200–250 g were chosen for the experiment and fasted overnight before to the trial. Rats were split at random into three groups, each containing six individuals (*n* = 6). The first group receives pure TNF solution (10 mg/kg body weight), the second group receives optimized SLN formulation TF10 (dosage equivalent to 10 mg/kg body weight), and the third group receives solvent control (sterile PB pH 7.4 solution). Using glass capillaries, blood samples (0.5 mL) were obtained from the retro-orbital plexus at predetermined intervals (0, 0.5, 1, 2, 3, 6, 12, 18, and 24 h). Upon collecting all of the samples into heparinized microtubes, they were centrifuged for 10 minutes at 5000 rpm at 4°C. 100 μL of leftover plasma was separated and analyzed by a standardized HPLC technique [67–69].

### 2.10. Stability Studies

A stability chamber (Inlab Equipments, India) was used to evaluate the stability of an optimized TNF-loaded SLN (TF10) formulation depending on %EE, average PS, and ZP. For 90 days, the SLN suspensions were packed in screw-capped amber-tinted glass bottles and stored at three distinct temperatures: 2–8°C, 25 ± 2°C/65 ± 5%RH, and 40 ± 2°C/75 ± 5%RH. The average particle diameter, ZP, and %EE of the samples were measured at predefined intervals (1, 30, 60, and 90 days). The results of these parameters before and after storage are contrasted [70–72].

## 3. Results and Discussion

### 3.1. Lipid Phase Screening

**Figure 2:**
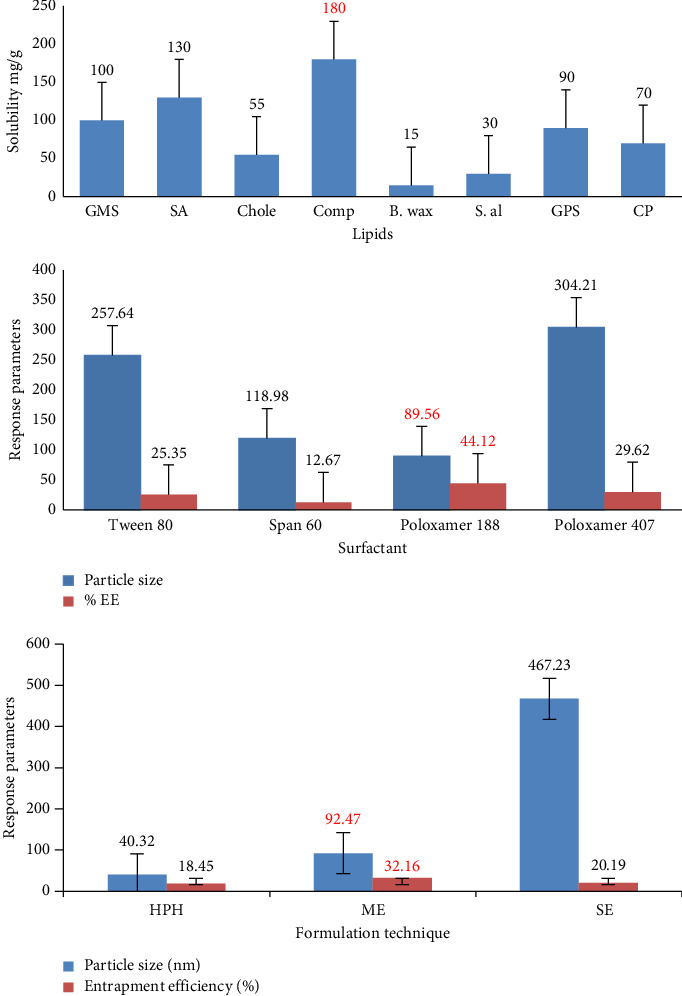
Screening of lipid, surfactant, and formulation technique (B wax = beeswax, Chole = cholesterol, Comp = Compritol 888 ATO, CP = cetyl palmitate, GMS = glyceryl monostearate, GPS = glyceryl palmitostearate, HPH = high-pressure homogenization, ME = microemulsion, SA = stearic acid, S. al = stearyl alcohol, and SE = solvent evaporation).

The drug's capacity to dissolve within a lipid matrix represents a critical parameter, given its influence on both the drug loading capacity and stability of SLNs. The solubility of the drug was assessed across eight different lipids: GMS, SA, cholesterol, Compritol, beeswax, stearyl alcohol, GPS, and CP, with the results depicted in Figure 2. TNF exhibited solubility in the following order: Compritol > SA > GMS > GPS > CP > cholesterol > stearyl alcohol > beeswax.

Among the lipids examined, Compritol 888 ATO demonstrated the highest solubility for TNF, with 180 mg of TNF solubilized per 1 g of lipid. SA exhibited the next greatest solubilizing capacity, with 130 mg of TNF solubilized per 1 g of lipid. Compritol 888 ATO is a class of acylglycerols derived from behenate and is comprised of a combination of three glycerides: behenic acid mono-, di-, and triglycerides. This lipid forms less ordered crystals, providing more space to accommodate larger quantities of drugs [73]. This characteristic may account for its notable solubility toward TNF, as confirmed by tests for %EE. Consequently, the lipid phase employed in the subsequent formulation of TNF-loaded SLNs was determined to be Compritol 888 ATO.

### 3.2. Screening of Surfactant

Surfactant plays a crucial role in stabilizing SLNs in aqueous environments [74]. A comprehensive screening of various surfactants, namely, Span 60, Tween 80, poloxamer 188, and poloxamer 407, was conducted. The outcomes of this surfactant assessment, depicted in Figure 2, were based on the average particle diameter and %EE. The surfactants were ranked according to reduced PS and augmented EE, with the order being poloxamer 188 > Tween 80 > poloxamer 407 > Span 60.

SLNs formulated with poloxamer 188 exhibited the smallest particle diameter and the highest EE among all tested surfactants. This outcome may be attributed to the comparatively lower molecular weight of poloxamer 188 in comparison with poloxamer 407, as well as its superior HLB value relative to other surfactants. The inherent hydrophilic properties of TNF (log P −1.6) [6] likely contributed to the enhanced entrapment when using poloxamer 188 as a surfactant. As a result, the GRAS status of poloxamer 188 [75] further justified its selection as the preferred surfactant for the subsequent formulation of TNF-loaded SLNs.

### 3.3. Screening of Formulation Technique

In a research context, SLN formulations were generated through different methods, namely, solvent evaporation, microemulsion, and HPH. The average PS and drug entrapment efficacy (EE) were evaluated for each technique, and the outcomes are graphically represented in Figure 2. Notably, the microemulsion approach yielded NPs characterized by diminutive dimensions and high drug EE. Given the hydrophilic nature of TNF, the microemulsion technique presents a distinct advantage in facilitating elevated drug loading [6, 76]. On the other hand, the HPH technique yielded nanometer-sized particles but with reduced EE, which may be attributable to the potential disruption of the outer stabilizer coat and lipid core, subsequently leading to drug leaching [77]. The use of organic solvents in the solvent evaporation process poses a significant toxicological challenge [78]. Consequently, the microemulsion approach is deemed the optimal method for the generation of TNF-loaded SLNs, characterized by its simplicity, reliability, and suitability for laboratory-scale production. Consequently, subsequent investigations were conducted utilizing the microemulsion technique.

### 3.4. Drug and Excipient Compatibility Study

**Figure 3:**
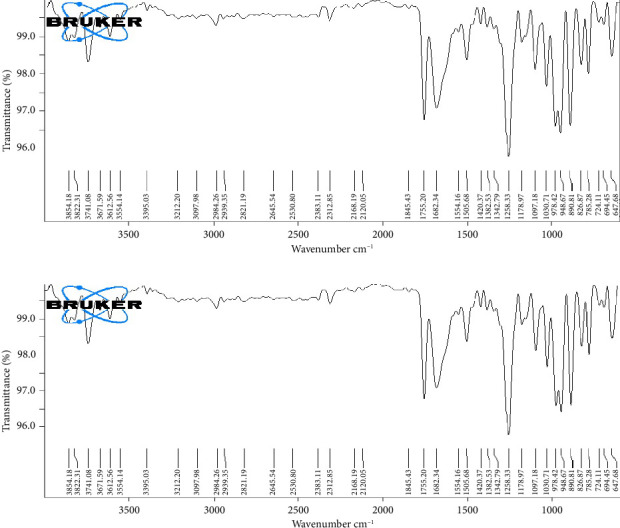
FTIR spectral analysis: (a) IR spectra of drug (tenofovir) and (b) IR spectra of physical mixture (drug (tenofovir) + lipid (Compritol 888 ATO) + surfactant (poloxamer 188).

The FTIR spectra depicting the pure drug TNF and the physical mixture are presented in Figure 3. The significant peaks observed in the IR spectra of TNF are at 3741 cm^−1^ (O-H stretching), 2984 cm^−1^ (C-H stretching, alkane), 1755 cm^−1^ (C=O stretching, ester), 1258 cm^−1^ (C-O stretching, alcohol), and 785 cm^−1^ (C-H bending). Upon comparison of the IR spectra of the drug and physical mixture, it was noted that there were no observable shifts or loss of functional peaks, indicating the absence of any substantial reaction between the drug (TNF) and the chosen excipients.

### 3.5. Method of Preparation

As per the experimental design outlined in Table 2, 13 formulations of TNF-loaded SLNs were prepared following the specified procedure. Subsequently, these formulations underwent evaluation for particle diameter, PDI, ZP, and %EE.

### 3.6. Characterization of Formulated TNF-Loaded SLNs

#### 3.6.1. PS, PDI, and ZP

The data presented in Table 3 pertain to the PS, PDI, and ZP of SLNs encapsulating TNF. It is observed that the average PS, PDI, and ZP of all 13 formulations ranged between 79.09 and 993.84 nm, 0.151 and 0.580, and −5.74 and −24.09 mV, respectively. Notably, despite all SLN dispersions exhibiting an average PS below 1000 nm, the sizes varied depending on the concentration of lipid and emulsifier. Moreover, all formulations demonstrated low PDI values, indicative of a narrow PS distribution. Furthermore, owing to the specific chemical composition of the lipid and surfactant employed, all SLN dispersions showcased a negative ZP, signifying the formulation's stability against aggregation.

When transporting SLNs, it is advisable to utilize the lymphatic system and ensure that their size falls within the range of 0.3–1.0 μm [79, 80]. A crucial aspect of formulation stability lies in the ZP, with a minimum ZP of ±20 mv being sought for maximal NP stability [24]. Enhanced ZP values yield stronger repulsive forces, thereby deterring particle aggregation and bolstering system stability. The PDI assesses the PS distribution around the mean, and a PDI value of less than 0.5 is deemed optimal for NPs [32]. These considerations warrant attention when choosing an optimized formulation. PS specifications for the majority of formulations indicated dimensions of less than 600 nm, signifying that the resulting SLNs were well suited for lymphatic transit, potentially accounting for heightened bioavailability. The NPs demonstrated exceptional stability and exhibited narrow size dispersion, as indicated by a PDI of less than 0.5 and ZP values exceeding 15 mV.

### 3.7. Effect of Variables on PS, PDI, and ZP

#### 3.7.1. Effect of Lipid Concentration

The concentration of lipids directly influences the size of particles, with PS increasing in direct proportion to the lipid concentration. Notably, formulations with higher lipid content (TF3, 7, 8, and 13) exhibit larger PSs (ranging from 600 to 1000 μm) compared to formulations with lower lipid content (TF2, 4, 9, and 12), which display PSs ranging from 10 to 100 μm. The study posits that in SLN formulations, PS escalates with heightened lipid concentration, potentially attributed to the notable interfacial tension between the aqueous and lipid phases, causing globules to coalesce and consequently yielding larger PSs [52]. Observations of the PDI values suggest that the lipid concentration has no discernible impact on the PDI values of the various formulations under investigation. Alternatively, the measured ZP values indicate that an elevation in the lipid concentration corresponds to an increase in ZP, likely due to heightened interfacial tension and increased ester groups of lipids associated with amplified lipid load [70].

#### 3.7.2. Effect of Surfactant Concentration

The PS is notably affected by the quantity of surfactant employed. A substantial reduction in PS is observed with an increase in surfactant content to 2%w/v in formulations TF3, 5, 11, and 12 and TF2, 4, 7, and 10. However, a contrary effect is noted with a further increase in surfactant concentration (3%w/v) in TF1, 6, 8, 9, and 13, resulting in an escalation in particle dimensions due to micelle generation [81]. The PDI exhibits a concurrent rise with surfactant concentration, which may be attributed to decreased surface tension, facilitating particle partitioning during homogenization. Notably, the ZP appears unaffected by the surfactant concentration.

#### 3.7.3. Effect of Homogenization Time

An increase in homogenization time was shown to result in a decrease in PS. This is explained by the force of droplet deformation increasing with larger homogenization cycles. Batches SLN1–SLN13 were created by varying the stirring time from 30 to 90 min. It was found that when homogenization time was increased from 30 to 60 min, the PS decreased gradually (TF4, 6, 7, and 11 and TF3, 8, 9, 10, and 12). Further increases in homogenization time (up to 90 min) (TF1, 2, 5, and 13) had no effect. However, the PDI increased with an increase in homogenization time. This may be due to the formation of foam at higher homogenization cycles. Moreover, homogenization for a longer time may lead to particle destabilization, resulting in particle aggregation and an increase in PDI values [70]. It may be inferred from the observed values of ZP that there was no significant relationship between homogenization time and ZP.

### 3.8. EE

The EE determinations are summarized in Table 3. The SLN dispersions loaded with TNF exhibited EEs ranging from 22.24% to 83.93%.

#### 3.8.1. Effect of Variables on EE

The study revealed that higher levels of lipids in the formulation were associated with increased EE. This phenomenon can be attributed to the heightened viscosity, which restricts drug migration into the surrounding dispersion medium. Additionally, a higher lipid concentration provides greater capacity to accommodate the drug, resulting in enhanced EE. However, exceeding a lipid concentration of 5% was found to diminish EE. Notably, formulations TF3, 7, 8, and 13, containing 10% w/v of lipids, exhibited reduced EE, likely due to expeditious lipid solidification and the subsequent partitioning of the hydrophilic drug TNF from the lipid matrix [82].

The addition of surfactant to SLN formulations has been reported to significantly enhance EE, particularly for hydrophilic drugs. The analysis of the data presented in Table 3 reveals a notable increase in EE from 70.90% to 81.55% with a substantial rise in the surfactant concentration. This enhancement is attributed to improved drug solubilization and its strong interaction with the internal lipid phase. However, when the surfactant concentration exceeds 2%w/v, the drug may form a micellar solution, leading to decreased entrapment percentage [81, 82]. Furthermore, extended homogenization times are observed to diminish EE due to the potential distortion of the outer stabilizer coat and lipid core, causing the drug to leach into the external phase [70].

The investigated factors, including lipid and surfactant concentrations, as well as homogenization time, yield a significant impact on dependent variables such as particle dimensions and %EE. A marked reduction in PS is associated with decreased lipid and increased surfactant concentrations and longer homogenization times. Conversely, elevated percentage entrapment is linked to higher lipid and surfactant concentrations and reduced homogenization time.

### 3.9. Data Analysis for Optimization

The empirical data were scrutinized by applying various models to the observed responses of 13 formulations using the STAT-EASE 360 trial version. After careful examination, it was determined that quadratic models provided the most accurate representation for the analyzed responses, particularly in relation to mean PS and %EE. Subsequently, the generated quadratic equations for the diverse responses are documented as follows:

(3) $$\begin{array}{c} \text{particle size}=+449.90+360.66A-9.69B+7.78C-52.55\;B-0.1750\;C+2.27\;C-42.50^{2}+85.35^{2}+2.37C^{2},\\ \%\text{entrapment efficiency}=+81.55+21.90A+3.80B-4.81C-6.70\text{AB}-1.24\text{AC}-1.37\text{BC}-18.79A^{2}-6.37B^{2}-5.91C^{2}, \end{array}$$

where A, B, and C represent the codified values for lipid level, surfactant quantity, and homogenization time, respectively.

Tables 4a and 4b present the ANOVA findings for the %EE and PS, respectively. Model variables A (lipid concentration), B (surfactant concentration), and C (homogenization duration) are significant, according to the ANOVA table for PS, which also shows that the model *F* value of 14.58 is significant with a *p* value of less than 0.05. However, there was no significance found for the model variables AB, AC, BC, A2, B2, and C2. Model terms A (lipid concentration), B (surfactant concentration), C (homogenization duration), AB, A2, B2, and C2 are significant, according to the ANOVA table for %EE, where the model F value of 86.9 is significant with a *p* value of less than 0.05. Model terms AC and BC, however, had no significance. PS and % EE had *R*^2^ values of 0.9106 and 0.9847, respectively, in Table 5, demonstrating a high association between the formulation factors and response parameters.

#### 3.9.1. Response Surface Plots

**Figure 4:**
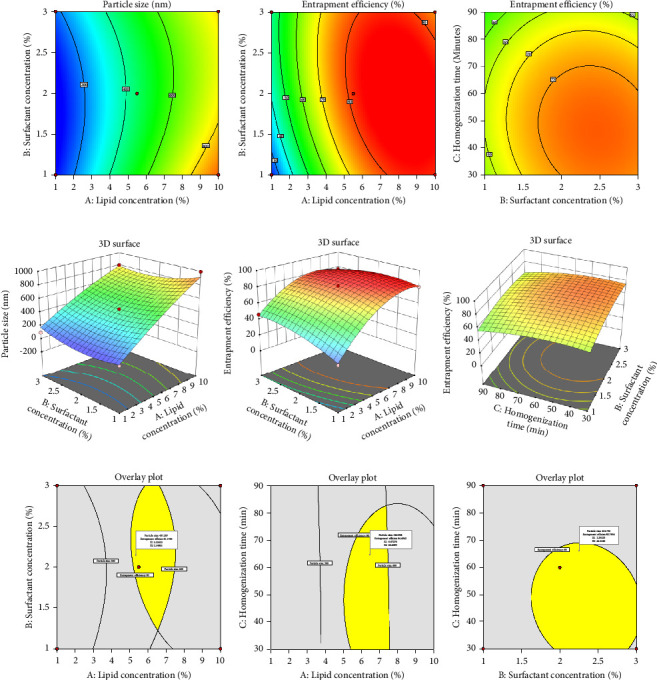
Contour and 3D surface graphs: (a–c) contour plots; (d–f) 3D surface graphs demonstrating the impact of independent parameters on Response I (particle size) and Response II (% entrapment efficiency); (g–i) overlay plots for optimization of TNF-loaded SLNs.

Contour and response surface models were utilized to elucidate the correlation between dependent and independent variables, as depicted in Figure 4. The analysis revealed the statistically significant influence of lipid concentration, surfactant content, and homogenization duration based on the 3D surface plots. The findings indicate that elevating lipid concentration positively influences PS and % EE, resulting in larger PS and higher % entrapment. Conversely, the increased surfactant content yields smaller PS and increased entrapment. Notably, the homogenization duration was found to have negligible effects on PS or % entrapment.

According to the Design-Experiment software, the most optimal formulation for achieving a high% EE and low mean PS entails utilizing a lipid concentration of 5.5%, a surfactant concentration of 2%, and a homogenization period of 60 min. In addition, a verification run was conducted to ensure the statistical dependability.

#### 3.9.2. Experimental Validation of Design Space

The superimposed plots depicted in Figure 3 delineate the design space and optimization parameters as prescribed by the DOE methodology to achieve specified targets. Batches were systematically manufactured and subjected to analysis for the determination of particle diameter and percent entrapment based on the defined parameters (illustrated in Figure 3). Remarkably, the values obtained from the batches closely approximated the projected values, exhibiting minimal bias (+0.637 for PS and +0.730 for percent entrapment, respectively). This convergence indicates the robustness and dependability of the optimization protocol.

### 3.10. Drug Release Studies

**Figure 5:**
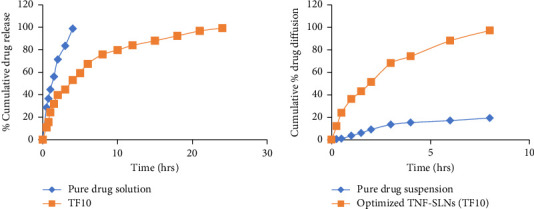
Comparing in vitro release and ex vivo permeation profiles of optimized SLNs formulation (TF10) with pure drug solution.

Figure 5 illustrates the in vitro release profile of pure TNF solution and various TNF-loaded SLN formulations. The cumulative release of TNF after 24 h ranged from 71.87% to 99.68% for the SLN dispersions, whereas the pure drug solution achieved nearly complete drug release within 4 h. Notably, TNF release from SLN dispersions exhibited a biphasic pattern, characterized by an initial burst release of approximately 24.38%–40.06% within the first 4 hours, followed by a sustained release. The study findings emphasize that higher lipid content in the SLNs is associated with delayed medication release, potentially offering extended therapeutic benefits. The initial burst release within four hours is postulated to result from drug adsorption on the surface of the SLNs, while the subsequent steady release for up to 24 hours is attributed to a diffusion mechanism slowly releasing the solubilized drug from the lipid matrix [83].

### 3.11. Release Kinetics

The in vitro release profiles were assessed using various kinetic models including zero-order, first-order, Higuchi, and Korsmeyer–Peppas models to characterize the release behavior. The determination of the goodness of fit for each model was based on the correlation coefficient (*R*^2^) values, with the model exhibiting the highest *R*^2^ value considered to be most representative of the release kinetics. It was observed that all formulations displayed a higher correlation coefficient for the first-order model, followed by the Higuchi model. These findings suggest that drug diffusion from the lipid matrix governs the release of the drug from SLNs. Additionally, the Korsmeyer–Peppas model revealed a release exponent (*n*) value smaller than 0.5, indicating that TNF is released from SLNs via Fickian diffusion.

### 3.12. Solid-State Characterization

#### 3.12.1. DSC

**Figure 6:**
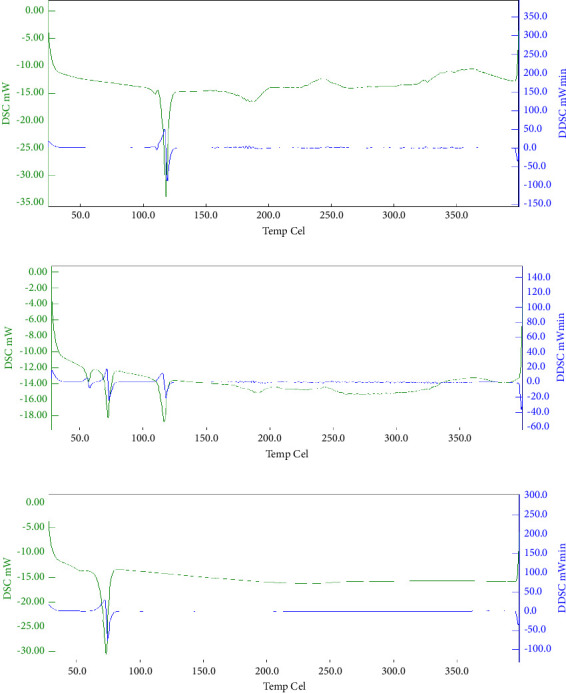
Differential scanning calorimetry graphs. (a) Pure drug tenofovir; (b) physical mixture of TNF + excipients; (c) optimized TNF-loaded SLN formulation (TF10).

DSC thermographs of TNF, a physical composite of TNF and Compritol 888 ATO, and the optimized TNF-loaded SLN formulation (TF10) are displayed in Figure 6. TNF has a prominent endothermic peak at 120°C on the DSC thermograph, confirming its melting point. The DSC thermograph for the physical mixture (TNF + Compritol 888 ATO) showed two endothermic maxima at 72°C and 120°C, correlated to the melting temperatures of TNF and Compritol 888 ATO, respectively. The appearance of two distinct peaks for the physical combination and no alteration in the endothermic peak of TNF show that TNF and the lipid (Compritol 888 ATO) have no chemical interactions. The DSC thermograph for optimized TNF-loaded SLNs shows only one endothermic peak at 72°C for Compritol 888 ATO, and the peak for pure drug TNF disappeared. This indicates that TNF is molecularly entrapped in the lipid matrix.

#### 3.12.2. PXRD

**Figure 7:**
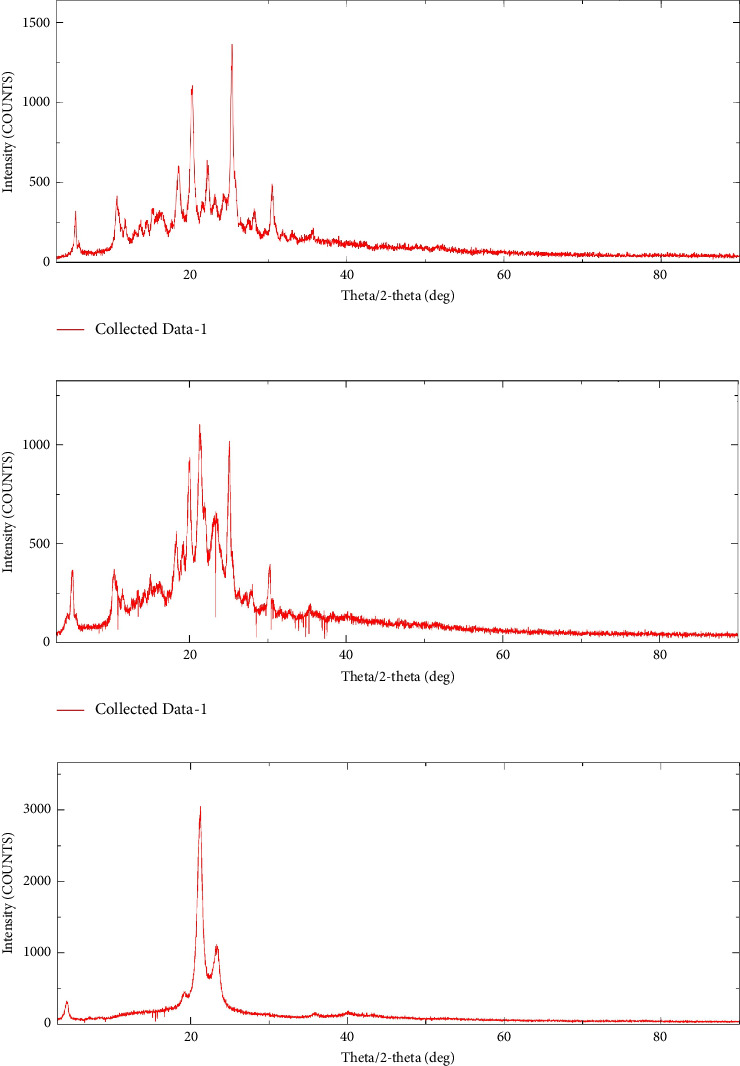
X-ray diffraction graphs. (a) Pure drug tenofovir; (b) physical mixture of TNF + excipients; (c) optimized TNF-loaded SLN formulation (TF10).

The PXRD spectra of TNF, a physical mixture of TNF and Compritol 888 ATO, and optimized TNF-loaded SLN formulation (TF10) are shown in Figure 7. The x-ray diffractogram of pure TNF showed strong peaks, indicating its crystalline nature. These peaks were likewise present with the same strength in the physical mixture but not in the TNF-loaded SLN diffractogram. This implies that the medication is totally miscible with the lipid matrix (Compritol 888 ATO) at the molecular level.

#### 3.12.3. SEM

**Figure 8:**
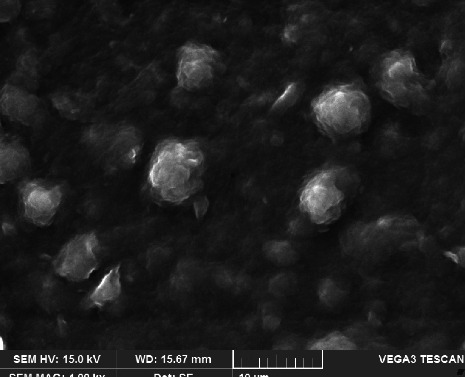
Scanning electron micrographs of optimized TNF-loaded SLN formulation (TF10).

The SEM analysis was applied to examine the dimensions and surface texture of optimized TNF-loaded SLNs. In Figure 8, SEM photomicrographs show that the formulated SLNs have a uniform size, smooth surface morphology, and spherical shape. The particles have a sealed SLN structure and no visible aggregation.

### 3.13. Ex Vivo Permeation Studies

The comparison of TNF's permeation profile is depicted in Figure 4, contrasting a pure drug suspension and TNF-loaded SLNs across a goat intestinal section. The ex vivo diffusion study, conducted for a duration of 8 h, unveiled that merely 19.36 ± 1.31% of the drug diffused from the TNF suspension, while an impressive 97.15 ± 0.90% of the drug permeated from the TNF-loaded SLNs. A notable outcome arising from the ex vivo permeation studies indicated that the apparent permeability coefficient (Papp) of TNF-loaded SLNs stood at 0.176 × 10^−6^ cm/s, a significant increase compared to the 0.012 × 10^−6^ cm/s Papp of the TNF suspension. The hydrophobic nature of the lipid matrix, in which the medication is enclosed, facilitates transport through the microfold cells within the intestinal epithelium, elucidating the considerable enhancement in the permeability of TNF-loaded SLNs in contrast to the bulk drug [48].

### 3.14. In Vivo Bioavailability Study

**Figure 9:**
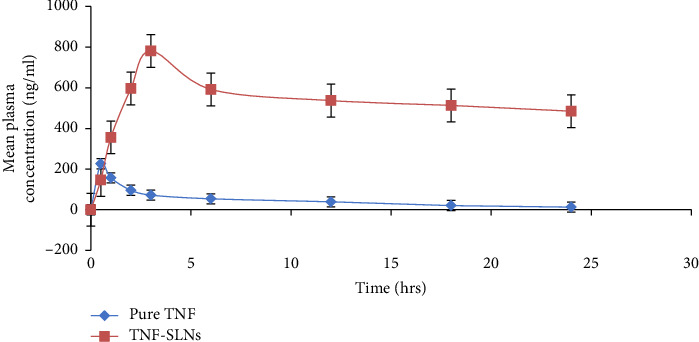
Tenofovir's plasma concentration vs time profile following oral administration of TNF-loaded SLNs and pure TNF solution (10 mg/kg).

In Figure 9, the oral pharmacokinetic properties of pure TNF and TNF-loaded SLNs are compared. The area under the curve (AUC), maximum drug levels in plasma (*C*_max_), and the duration to reach peak level (*T*_max_) were computed utilizing the linear-log trapezoidal approach based on the plasma concentration (Cp) vs. time profile graph. The results in Table 6 indicate that *T*_max_, *C*_max_, *K*_*a*_, *t*_1/2_, mean residence time (MRT), and AUC values were notably higher with TNF-loaded SLNs as compared to pure TNF, signifying a substantial increase in oral bioavailability. Despite ex vivo permeation experiments demonstrating elevated permeability, this does not necessarily correlate to improved drug absorption. As a result, in vivo pharmacokinetic experiments were conducted, confirming a 12.4-fold increase in bioavailability compared to the pure TNF solution. The amplified bioavailability of TNF-loaded SLNs could be attributed to lymphatic uptake by M cells in Peyer's patch, improved penetration across the gastrointestinal (GI) membrane, inhibition of P-gp efflux transport, and increased paracellular transport induced by the lipid matrix [48, 84].

### 3.15. Stability Studies

The stability of optimized TNF-loaded SLN formulation was tested under various storage conditions, and the findings are reported in Table 7. At 25 ± 2°C/65 ± 5%RH and 2–8°C, no changes in physical appearance, particle dimensions, % EE, and ZP were noted during 3 months. At 40 ± 2°C/755%RH, there had been a modest rise in the particle diameter and a drop in EE although this may not be relevant. These findings show that the formulation produced is stable and has a long shelf life.

## 4. Conclusion

The study delineated the advantages of systematically screening lipid, surfactant, and formulation techniques to enhance the loading capacity of hydrophilic drugs in SLNs. The challenge of encapsulating hydrophilic drugs within the hydrophobic matrix of SLNs, attributed to these drugs' tendency to migrate into the aqueous phase during the manufacturing process, was addressed. The study successfully developed SLNs loaded with the hydrophilic antiviral drug TNF (BCS Class III) using the microemulsion process with Compritol as the lipid, employing the BBD. The resulting SLNs demonstrated a PS of 449.90 ± 4.79 nm and an EE of 83.13 ± 6.34%. The investigation disclosed a 12.4-fold increase in bioavailability for TNF-loaded SLNs compared to pure TNF solution, as observed in albino Wistar rats. This suggests that SLNs could serve as carriers to enhance the bioavailability of BCS Class III medicines (hydrophilic pharmaceuticals), considering their limited oral bioavailability attributed to permeability constraints.

### 4.1. Future Perspective

The researchers contemplate assessing the in vivo performance of TNF-loaded SLNs to evaluate the formulation's potential in managing HIV infections. Furthermore, they plan to investigate the influence of surface charge on SLN lymphatic absorption. This groundbreaking discovery is anticipated to offer a platform technology for augmenting the delivery of therapeutic compounds demonstrating low bioavailability due to limited permeability, gut metabolism, and P-gp efflux transport, particularly antiviral medications.

## Figures and Tables

**Table 1:** Components of quality target product profile (QTPP) and critical quality attributes (CQAs) for optimizing tenofovir-loaded solid lipid nanoparticles (TNF-loaded SLNs).

	Target	Justification
*Quality target product profile (QTPPs)*		
Drug delivery system	Solid lipid nanoparticles	Promotes oral bioavailability and greater brain uptake
Dosage type	Controlled release	Prolonged drug therapy is achieved
Route of administration	Oral	Patient compliance
Drug release	More than 80%	Necessary for the best possible treatment outcome

*Critical quality attributes (CQAs)*		
Particle size	Less than 200 nm	Offers lymphatic uptake and escapes reticular endothelial system (RES)
Entrapment efficiency	More than 80%	Enhanced pharmacological and therapeutic action

**Table 2:** Experimental runs and coded values for optimizing TNF-loaded SLNs using the Box–Behnken design.

No. of runs	Formulation code	Factor 1 (A: lipid, %w/v)	Factor 2 (B: surfactant, %w/v)	Factor 3 (C: homogenization time, min)
1	TF1	5.5	3	90
2	TF2	1	2	90
3	TF3	10	1	60
4	TF4	1	2	30
5	TF5	5.5	1	90
6	TF6	5.5	3	30
7	TF7	10	2	30
8	TF8	10	3	60
9	TF9	1	3	60
10	TF10	5.5	2	60
11	TF11	5.5	1	30
12	TF12	1	1	60
13	TF13	10	2	90

**Table 3:** Resulting data from mean particle size, PDI, zeta potential, and % entrapment efficiency analysis of TNF-loaded SLNs.

Formulation code	Particle size (Response 1) (nm)	PDI	Zeta potential (mV)	Entrapment efficiency (Response 2) (%)
TF1	573.31 ± 2.34	0.580 ± 0.163	−17.72 ± 2.34	58.16 ± 4.22
TF2	99.92 ± 4.23	0.498 ± 0.176	−10.32 ± 3.91	34.67 ± 2.07
TF3	993.84 ± 4.56	0.527 ± 0.008	−21.43 ± 2.34	76.54 ± 3.12
TF4	86.71 ± 6.45	0.151 ± 0.058	−8.78 ± 2.38	40.05 ± 4.98
TF5	520.23 ± 2.87	0.563 ± 0.154	−14.19 ± 1.48	66.14 ± 1.82
TF6	550.57 ± 3.58	0.346 ± 0.47	−16.90 ± 3.27	75.34 ± 3.07
TF7	720.12 ± 4.89	0.393 ± 0.42	−22.68 ± 2.98	81.02 ± 4.12
TF8	801.40 ± 1.34	0.503 ± 0.006	−24.09 ± 1.19	76.10 ± 6.06
TF9	96.80 ± 2.67	0.298 ± 0.005	−7.45 ± 3.67	40.89 ± 5.19
TF10	449.90 ± 4.79	0.304 ± 0.004	−18.10 ± 2.35	83.13 ± 6.34
TF11	506.51 ± 5.94	0.314 ± 0.004	−13.70 ± 2.92	72.76 ± 3.29
TF12	79.09 ± 3.97	0.271 ± 0.012	−5.74 ± 3.56	24.78 ± 4.52
TF13	732.50 ± 2.65	0.549 ± 0.123	−23.02 ± 1.14	68.45 ± 4.86

**(a):** 

**Source**	**Sum of squares**	**df**	**Mean square**	***F* value**	**p** **value**	

Model	1.087*E* + 06	9	1.208*E* + 05	14.58	0.0247	Significant
A—lipid concentration	1.041*E* + 06	1	1.041*E* + 06	125.57	0.0015	Significant
B—surfactant concentration	750.78	1	750.78	0.0906	0.0310	Significant
C—homogenization time	483.60	1	483.60	0.0584	0.0247	Significant
AB	11,046.01	1	11,046.01	1.33	0.3319	Not significant
AC	0.1225	1	0.1225	0.0000	0.9972	Not significant
BC	20.70	1	20.70	0.0025	0.9633	Not significant
*A* ^2^	4128.57	1	4128.57	0.4982	0.5312	Not significant
*B* ^2^	16,650.57	1	16,650.57	2.01	0.2514	Not significant
*C* ^2^	12.89	1	12.89	0.0016	0.9710	Not significant
Residual	24,862.47	3	8287.49			—
Cor total	1.112*E* + 06	12				—

**(b):** 

**Source**	**Sum of squares**	**df**	**Mean square**	***F* value**	**p** **value**	

Model	5154.04	9	572.67	86.91	0.0018	Significant
A—lipid concentration	3836.44	1	3836.44	582.26	0.0002	Significant
B—surfactant concentration	115.82	1	115.82	17.58	0.0247	Significant
C—homogenization time	185.19	1	185.19	28.11	0.0131	Significant
AB	179.83	1	179.83	27.29	0.0136	Significant
AC	6.13	1	6.13	0.9297	0.4061	Not significant
BC	7.56	1	7.56	1.15	0.3625	Not significant
*A* ^2^	806.90	1	806.90	122.46	0.0016	Significant
*B* ^2^	92.78	1	92.78	14.08	0.0331	Significant
*C* ^2^	79.80	1	79.80	12.11	0.0401	Significant
Residual	19.77	3	6.59			—
Cor total	5173.81	12				—

**Table 5:** Summary of Box–Behnken design's ANOVA results for TNF-loaded SLN optimization.

S. no.	Parameter	Response I (particle size)	Response II (% entrapment efficiency)	Outcome
1	*F* value	14.58	86.91	Model terms were significant
2	*p* value	0.0247	0.0018	Model terms were significant
3	*R* ^2^ value	0.9106	0.9847	Response parameters and formulation variables exhibit good agreement

**Table 6:** Pharmacokinetic data of pure TNF solution and optimized TNF-loaded SLNs (TF10), mean ± SD (n = 6).

Parameter	Pure TNF solution	Opt. TNF-loaded SLNs (F7)
*C*_max_	226 ± 510.56 ng/mL	781 ± 4.12 ng/mL
*T*_max_	0.5 h ± 0.01	3 h ± 0.01
*K*_*a*_	0.43 ± 0.27 h^−1^	3.59 ± 2.17 h^−1^
*K*_*E*_	1.18 ± 0.49 h^−1^	0.57 ± 0.22 h^−1^
*t*_1/2_	10.6 ± 2.02 h	18.61 ± 0.35 h
AUC_0−24_	1237 ± 52.41 ng/h mL^−1^	15,338 ± 22.30 ng/h mL^−1^
[AUC]_0−*∞*_	1049.28 ± 155.16 ng/h mL^−1^	29,083.16 ± 118.36 ng/hr mL^−1^
MRT	16.28 ± 3.68 h	36.12 ± 2.12 h

**Table 7:** Stability study data of optimized TNF-loaded SLN (TF10) formulation.

Stability testing condition	Parameters	Sampling intervals			
		0 days	30 days	60 days	90 days
2–8°C	Particle size	449.90 ± 4.79	449.16 ± 5.04	449.78 ± 5.18	450.16 ± 5.04
	%EE	81.55 ± 1.74	81.53 ± 1.23	81.43 ± 1.57	81.32 ± 1.65
	Zeta potential	−18.10 ± 2.35	−18.10 ± 2.35	−18.10 ± 2.35	−18.10 ± 3.25

25 ± 2°C/65 ± 5%RH	Particle size	449.90 ± 4.79	449.97 ± 5.12	449.97 ± 6.09	452.17 ± 2.09
	%EE	81.55 ± 1.74	81.47 ± 1.06	81.36 ± 1.53	80.97 ± 1.04
	Zeta potential	−18.10 ± 2.35	−18.02 ± 2.54	−18.02 ± 3.07	−18.02 ± 3.07

40 ± 2°C/75 ± 5%RH	Particle size	449.90 ± 4.79	451.54 ± 5.03	451.54 ± 7.34	455.54 ± 3.17
	%EE	81.55 ± 1.74	81.37 ± 2.06	81.03 ± 3.19	80.03 ± 4.01
	Zeta potential	−18.10 ± 2.35	−18.01 ± 3.51	−17.01 ± 2.15	−17.01 ± 1.15

## Data Availability

The data that support the findings of this study are available from the corresponding author upon reasonable request.
